# P Wave Duration/P Wave Voltage Ratio Plays a Promising Role in the Prediction of Atrial Fibrillation: A New Player in the Game

**DOI:** 10.1155/2021/8876704

**Published:** 2021-05-29

**Authors:** E. Karacop, A. Enhos, N. Bakhshaliyev, R. Ozdemir

**Affiliations:** Bezmialem Foundation University, Faculty of Medicine, Department of Cardiology, Istanbul, Turkey

## Abstract

**Background:**

Atrial fibrillation (AF) is the most common sustained arrhythmia in clinical practice. Identification of patients at risk for developing AF and the opportunity for early targeted intervention might have a significant impact on morbidity and mortality. Prolonged P wave duration and decreased P wave voltage have been shown to be independent predictors of AF. The present study aimed to investigate the role of P wave duration/P wave voltage in predicting new-onset AF.

**Methods:**

We screened a total of 640 consecutive patients who admitted to cardiology outpatient clinic with a complaint of palpitation between 2012 and 2014. 24-h Holter monitoring, echocardiography, and electrocardiography (ECG) recordings were reviewed to identify new-onset AF. Patients were assigned into two groups based on presence (*n* = 150) and absence (*n* = 490) of new-onset AF. Previous ECGs with sinus rhythm were analyzed. P wave duration was measured in inferior leads, and P wave voltage was measured in lead one. P wave duration/P wave voltage was also calculated for each patient.

**Results:**

One hundred fifty subjects (23.4%) had new-onset AF among 640 patients. P wave duration (123.27 ± 12.87 vs. 119.33 ± 17.39 ms, *p*=0.024) and P wave duration/P wave voltage (1284.70 ± 508.03 vs. 924.14 ± 462.06 ms/mV, *p* < 0.001) were higher, and P wave voltage (0.12 ± 0.04 vs. 0.13 ± 0.04 mV, *p* < 0.001) was significantly lower in the new-onset AF group compared with non-AFs. P wave duration/P wave voltage, with a cut off of 854.5 ms/mV, had 83.3% sensitivity and 62.0% specificity in a receiver operating characteristic curve (AUC 0.728, 95% CI 0.687–0.769; *p* < 0.001). Their negative and positive predictive values were 78.7% and 68.6%, respectively. In a univariate regression analysis, age, smoking, C-reactive protein, brain natriuretic peptide, left atrial diameter, left atrial volume index, P wave duration, P wave voltage, and P wave duration/P wave voltage were significantly associated with the development of new-onset AF. Moreover, smoking (OR 4.008, 95% CI 1.707–9.409; *p*=0.001), left atrial volume index (OR 7.108, 95% CI 4.400–11.483; *p* < 0.001), and P wave duration/P wave voltage (OR 1.002, 95% CI 1.000–1.003; *p*=0.044) were found to be significant independent predictors of new-onset AF in a multivariate analysis, after adjusting for other risk parameters.

**Conclusion:**

The P wave duration/P wave voltage ratio is a practical, easy-to-use, cheap, and reliable electrocardiographic parameter, which can play a promising role for both in predicting and elucidating a mechanism of new-onset AF.

## 1. Introduction

Atrial fibrillation (AF) is the most commonly encountered sustained arrhythmia in clinical practice. It increases in prevalence with advancing age. Approximately 1% of patients with AF are <60 years of age, whereas up to 12% of patients with AF are 75 to 84 years of age [1]. Despite good progress in the treatment of AF, it remains one of the major causes of cardiovascular mortality and morbidity, including acute coronary syndromes, heart failure, and stroke in the world. Identification of patients at risk for developing AF is of paramount importance. The baseline electrocardiography (ECG) of patients prone to AF is an easily accessible noninvasive tool. It might show subclinical structural atrial abnormalities resulting from adverse remodeling of the atrial electroanatomic substrate [2, 3]. The P wave represents atrial depolarization and is generally accepted as the most reliable marker of atrial conduction.

Previous studies have postulated that there was an increased risk of AF with longer P wave duration [4]. Several cross-sectional studies have indicated that subjects with AF, on average, have longer P wave duration compared with healthy controls [5]. More recently, prolonged P wave duration has been shown to be a marker of incident AF in two independent cohort studies [6, 7]. Investigators from these studies speculated that prolonged atrial conduction time, represented by prolonged P wave duration, was an intermediate step in the accumulation of atrial insults, ultimately leading to AF. A duration of the P wave above 120 ms had been reported to be an independent predictor of AF [8].

Additionally, decreased P wave voltage in lead 1 was found to be correlated with massive fibrosis in the atrium of the heart [9]. Reduced P wave voltage was thought to mirror altered atrial conduction patterns seen in interatrial block (IAB). Left atrial voltage activation maps have shown that in patients with reduced P wave voltage, the electrical impulse in Bachmann's bundle region is displaced [10]. Recently, the morphology-voltage-P-wave duration (MVP) has been reported as an electrocardiographic risk score for predicting new-onset AF [11]. Alexander et al. [11] included P wave voltage, duration, and IAB in the MVP ECG risk score for prediction of AF. It allocated points according to presence of IAB, amplitude of voltage, and prolongation of duration. Inclusion of IAB and prolongation of P wave duration in the same scoring system became a debate since there were many intersection points among these parameters.

IAB was not included in our study. The main purpose of the study was to determine whether or not a P wave duration/P wave voltage ratio as an electroanatomical parameter in ECG recorded during sinus rhythm could identify patients with higher susceptibility to new-onset AF.

## 2. Materials and Methods

### 2.1. Study Population

1100 patients who admitted to cardiology outpatient clinic with a complaint of palpitation between 2012 and 2014 years were retrospectively screened. Among these, 460 patients were not included in this analysis due to the presence of exclusion criteria. Finally, 640 patients (mean age; 67.3 ± 7.0 years, 61.7% female) with a median 5-year screening were included in the study. Study groups were divided into two groups as follows: 150 patients with new-onset AF and 490 patients without AF.

Exclusion criteria of the study were as follows: previous AF on ECG recorded during the first visit in the outpatient clinic, lack of measurable P waves in lead 1 or in the inferior leads, second or third degree atrioventricular blocks, history of pacemaker implantation, class I and III antiarrhythmic usage, rheumatic heart disease, pericarditis, end-stage hepatic and renal failure (known chronic liver disease or aspartate aminotransferase > 3 times than normal range, stage 3 and 4 chronic kidney disease) and acute cardiovascular or cerebrovascular events within the preceding 3 months, hypo-hyperthyroidism, and any drug use for thyroid diseases.

The local ethics committee approved the study protocol, and the study was conducted in accordance with ethical principles described in the Declaration of Helsinki.

### 2.2. Data Collection, Follow-Up, and Definition

The following data of the study subjects were screened: (a) demographic and clinical characteristics (Table 1); (b) cardiovascular risk factors including hypertension, hyperlipidemia, and diabetes mellitus (Table 1); (c) cardiovascular disease history such as congestive heart failure, cerebral infarction or transient ischemic attack (TIA), and structural heart disease (Table 1). Cardiovascular status was determined for each patient by using ECG, transthoracic echocardiography (Table 2), biochemical and hematological measurements (Table 3), and 24-h Holter recordings (Table 4), in their initial visits. Drug use is shown in Table 5.

A standard 12-lead ECG or a single-lead tracing of ≥30 seconds showing heart rhythm with no discernible repeating P waves and irregular RR intervals is diagnostic of clinical AF. [12] Patient charts were reviewed for baseline demographic and clinical data. All available clinical records, ECGs, and Holter monitors were reviewed for the presence of AF. Patients were followed up until their last available clinic record. AF was diagnosed if found to be present on an ECG documented at a clinic visit, hospital records, or Holter reports. A follow‐up ECG was conducted at outpatient clinic within 5 years of the index ECG.

### 2.3. Laboratory Measurements

Fasting blood samples were taken from a large antecubital vein of each patient to determine biochemical and hemogram parameters. Ethylenediaminetetraacetic acid (EDTA) tubes were used for automatic blood count. Blood counts were measured with a Beckman Coulter LH 780 hematology analyzer. Total cholesterol, triglyceride, low-density lipoprotein cholesterol, and high-density lipoprotein cholesterol were measured by the colorimetric method (Abbott Laboratories, Abbott Park, IL, USA). Quantitative assessment of serum brain natriuretic peptide (BNP) was measured using a BNP chemiluminescent microparticle immunoassay, and samples were analyzed on an Abbott ARCHITECT analyzer (Abbott Diagnostics Division, Malvern, PA, USA). Serum C-reactive protein (CRP) levels were determined by turbidimetric immunoassay (CRP kit; Beckman Coulter), and the examinations were conducted in the Array 360 Analyzer (Beckman Coulter). Glucose, creatinine, and other parameters were determined by standard methods.

### 2.4. Electrocardiography

ECGs were performed with the GE MAC 3500 Resting ECG System (General Electric, Boston, MA) or previous models. Standard 12-lead ECG was recorded at 25 mm/s paper speed and 10 mm/mv amplitude in the supine position for each subject. Images were amplified ×8 and analyzed using ICONICO semiautomatic calipers. ECGs with sinus rhythm during the first visit in the outpatient clinic were analyzed from all participants. P wave duration was computed as the difference between the onset and the offset of P wave and was measured in inferior leads. P wave voltage was measured from the peak of the P wave to the isoelectric line of the T-P interval in lead 1. P wave duration/P wave voltage was calculated from each participant. Each ECG was blindly reviewed by two independent cardiologists. Interobserver and intraobserver variabilities were 2.8% and 2.3%, respectively.

### 2.5. Echocardiography

Echocardiography has a major role in the assessment of AF prediction as it provides insights into the cardiac function, etiology and diagnosis of the underlying structural heart disease, and risk stratification. Standard comprehensive transthoracic echocardiography was performed for each subject in their initial visits. Left chamber dimensions, left atrial volume index, wall thickness, ejection fraction, and pulmonary artery systolic pressure were measured. Valvular stenosis and regurgitation were graded according to current guidelines [13]. Echocardiographic assessment was performed using a VIVID 7 Dimension cardiovascular ultrasound system (Vingmed-General Electric, Horten, Norway) with a 3.5 MHz transducer. Left ventricle (LV) diameters were measured using M-mode imaging, and left atrial area was calculated in the four chamber apical view. Moreover, ejection fraction was calculated by using modified Simpson's method. All echocardiographic examinations were performed by an experienced cardiologist.

### 2.6. Statistical Analysis

Data analysis was performed by SPSS 17 (SPSS Inc., Chicago, Illinois, USA) package software. The Shapiro–Wilk test was used for assessing whether the variables follow normal distribution or not. Continuous variables are expressed as mean ± standard deviation (SD) and categorical variables as numbers and percentages. Differences between independent groups were assessed by unpaired Student's *t*-test for normally distributed quantitative variables and Mann-Whitney's *U* test for variables without normal distribution and Chi-square for qualitative variables. Pearson correlation analysis was performed for correlation between P wave duration/P wave voltage and CRP and BNP. An exploratory evaluation of additional cut-points was performed using the receiver operating characteristic (ROC) curve analysis. Moreover; sensitivity, specificity, and negative and positive predictive values for new-onset AF were also calculated. Univariate and multivariate logistic regression analyses were done to determine significant independent predictors of new-onset AF. A *p* value < 0.05 was considered statistically significant. All *p* values were two-sided.

## 3. Results

The study population consisted of 640 patients with a mean age of 67.3 ± 7.0 years. Baseline demographic and echocardiographic characteristics of the study group are presented in Tables 1 and 2. The population was predominantly female (61.7%). The incidence of new-onset AF was 23.4% during a mean follow-up of 240 weeks. The average time of AF occurrence was 273 weeks (95% CI 247–298 weeks). Patients were assigned into two groups based on presence (*n* = 150) and absence (*n* = 490) of new-onset AF. There was no difference between two groups in terms of age, gender, body mass index, hypertension, hyperlipidemia, diabetes mellitus, past history of cerebrovascular event, heart failure, and congestive heart failure-hypertension-age ≥ 75 years-diabetes mellitus-stroke-vascular disease-age 65–74 years-sex (CHA_2_DS_2_VASc) score. Smoking status (44.7 vs. 30.0%; *p*=0.001) was higher in the new-onset AF group than non-AFs. As it was expected, patients who developed AF had a significantly higher left atrial volume index (36.0 ± 1.5 vs 31.2 ± 1.8 mL/mm^2^; *p* < 0.001).

**Figure 1:**
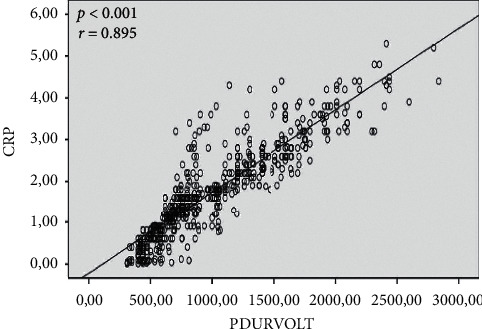
Correlation between P wave duration/P wave voltage and CRP. There is a significant correlation between P wave duration/P wave voltage and CRP (*r* = 0.895, *p* < 0.001). CRP: C-reactive protein.

**Figure 2:**
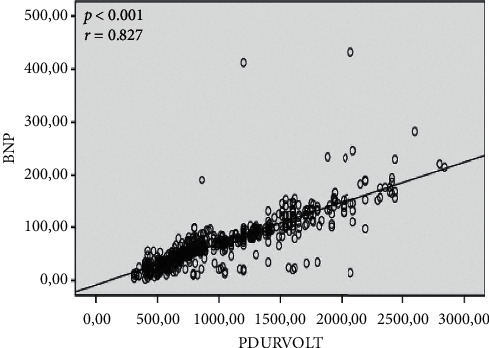
Correlation between P wave duration/P wave voltage and BNP. There is a significant correlation between P wave duration/P wave voltage and BNP (*r* = 0.827, *p* < 0.001). BNP: brain natriuretic peptide.

Laboratory findings are summarized in Table 3. CRP (2.3 ± 1.0 vs. 1.5 ± 1.0 pg/mL; *p* < 0.001) and BNP (92.3 ± 56.4 vs. 62.8 ± 40.6 pg/mL; *p* < 0.001) were higher in the new-onset AF group. There was a significant correlation between P wave duration/P wave voltage and CRP (*r* = 0.895, *p* < 0.001) and BNP (*r* = 0.827, *p* < 0.001) (Figures 1 and 2).

**Figure 3:**
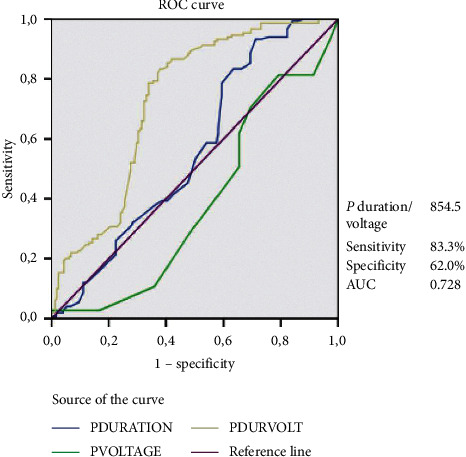
P wave duration/P wave voltage cutoff of 854.5 predicts new-onset AF, with a sensitivity of 83.3% and a specificity of 62.0%. The AUC of P wave duration is 0.561. The AUC of P wave voltage is 0.387. The AUC of P wave duration/P wave voltage ratio is 0.728. AF: atrial fibrillation, AUC: area under the curve, ROC: receiver operating characteristic. Diagonal segments are produced by ties.

P wave duration (123.2 ± 12.8 vs. 119.3 ± 17.3 ms, *p*=0.024) and P wave duration/P wave voltage (1284.7 ± 508.0 vs. 924.1 ± 462.0 ms/mV, *p* < 0.001) were significantly higher in subjects with new-onset AF. However, P wave voltage (0.12 ± 0.04 vs. 0.13 ± 0.04 mV, *p* < 0.001) was found to be lower due to fibrosis in the new-onset AF group (Table 6). A receiver operating characteristic (ROC) curve was generated to determine sensitivity and specificity, and the respective areas under the curve (AUCs) were used to investigate the predictive value of P wave duration/P wave voltage for the new-onset AF group (Figure 3). The analysis indicated that P wave duration/P wave voltage > 854.5 ms/mV had a 83.3% sensitivity and 62.0% specificity (AUC 0.728, 95% CI 0.687–0.769; *p* < 0.001). Moreover, negative and positive predictive values were 78.7% and 68.6%, respectively.

In a univariate regression analysis, age, smoking, CRP, BNP, left atrial diameter, left atrial volume index, P wave duration, P wave voltage, and P wave duration/P wave voltage were significantly related with the development of new-onset AF. Furthermore, smoking (OR 4.008, 95% CI 1.707–9.409; *p*=0.001), left atrial volume index (OR 7.108, 95% CI 4.400–11.483; *p* < 0.001), and P wave duration/P wave voltage (OR 1.002, 95% CI 1.000–1.003; *p*=0.044) were found to be significant independent predictors of new-onset AF in a multivariate analysis, after adjusting for other risk parameters (Table 7).

## 4. Discussion

In this study, we assessed both the relationship and predictive role of the P wave duration/P wave voltage ratio with presence of new-onset AF. The main findings of the study were as follows:

1. P wave duration and P wave duration/P wave voltage were found to be significantly increased and P wave voltage decreased in the new-onset AF group
2. P wave duration/P wave voltage > 854.5 ms/mV could predict new-onset AF with a sensitivity of 83.3% and specificity of 62.0%
3. Smoking, left atrial volume index, and P wave duration/P wave voltage ratios were important independent predictors of new-onset AF in a multivariate analysis

Noninvasive predictors of AF including age and left atrial size on echocardiography had been identified in previous studies [14–16]. Premature atrial contraction on Holter monitoring had also been reported as a strong finding, which were significantly related with occurrence of AF [17, 18]. 12-lead resting ECG—with the features of low cost, practical, and easy to use—remains the most frequently used screening and diagnostic tool for the evaluation of patients in terms of cardiovascular disease. The P wave represents atrial depolarization and is an electrocardiographic marker of atrial conduction. One population-based study identified P wave morphology as one of the very strong predictors of AF [19]. Recently, the morphology-voltage-P-wave duration (MVP) has been reported as an electrocardiographic risk score for predicting new-onset AF [11]. Our study was the first assessing the combination of P wave duration and voltage, indicated by P wave duration/P wave voltage ratio, as a novel parameter to discriminate patients prone to new-onset AF.

A potential increased thromboembolic risk of asymptomatic AF compared to symptomatic ones were reported in both the mode selection trial (MOST) and the relationship between daily atrial tachyarrhythmia burden from implantable device diagnostics and stroke risk studies (TRENDS) [20–23]. A recent expert opinion has confirmed that incidentally detected AF has not benign setting and justifies consideration of anticoagulation in those with stroke risk factors [24]. As a result of commercially available hand-held cardiac rhythm recorders development, someone can record a rhythm strip by using smartphone technology [25]. Inclusion criteria for screening AF in the assessment of remote heart rhythm sampling using the AliveCor heart monitor to screen for atrial fibrillation (REHEARSE-AF) study were individuals > 65 years of age with a CHA_2_DS_2_-VASc score ≥ 2, who were not under oral anticoagulation therapy for lack known diagnosis of new-onset AF, well-known contraindication to anticoagulation, and permanent pacemaker implantation [26]. They postulated in their study that age was the strongest predictor of AF [27], and a screening age cutoff value of ≥65 years was recommended on the basis of current guidelines [28]. However, recently, no precise consensus has been provided, who may require screening with hand-held cardiac rhythm recorders. Furthermore, it is estimated that annual sales of such devices will reach 50 billion dollars worldwide in the future [29]. The ability of some devices to accurately measure biometric endpoints has been questioned, and some mobile health technologies are available without verification through rigorous clinical studies [30]. P wave duration/P wave voltage may help clinicians to decide which patients warrant further monitoring through these devices to detect new-onset AF.

IAB is caused by defects in the conduction through Bachmann's bundle. Partial IAB is defined as P wave duration > 120 ms with a normal P wave morphology in the inferior leads [31]. Advanced IAB is defined as P wave duration > 120 ms together with biphasic P waves with a negative terminal deflection below the isoelectric line in these leads [31]. Its prevalence increases with age reaching 60% in those who are >50 years of age [32]. A significant intersection between P wave duration, voltage, and IAB was reported in previous studies. In addition, partial IAB was found to be correlated with advanced fibrosis in the atrium like IAB [31–34]. While recent studies have reported an increase in the risk of developing new-onset AF when IAB is observed [33, 34], some studies did not support this association [35, 36]. In our study, P wave duration and P wave duration/P wave voltage were found to be significantly increased and reduction of P wave voltage was reported in the new-onset AF group. Moreover, a P wave duration/P wave voltage ratio > 854.5 ms/mV was able to predict new-onset AF with a high sensitivity of 83.3% and moderate specificity. However, IAB was not included in our study data, which may be a logical explanation for moderate specificity of P wave duration/P wave voltage ratio in this setting.

Alexander et al. [11] included P wave voltage, duration, and IAB in the MVP ECG risk score for prediction of AF. It allocated points according to presence of IAB, amplitude of voltage, and prolongation of duration. The mean axis of atrial depolarization was the spatial sum of multiple vectors and was expressed as a vector directed to the left, downward, and forward [37]. This vectors spread in three-dimensional direction. The scoring systems including P wave duration and voltage to predict atrial fibrillation were electroanatomical rather than clinical. Additionally, the power of these parameters was higher than the clinical parameters. We hypothesized and found that the P wave duration/P wave voltage ratio is a novel practical parameter, which would provide enough power for screening patients prone to new-onset AF.

This study has several limitations. Although sample size is large enough, the study is single-centered and has a retrospective design. A prospective multicenter study may allow better assessment of predictive values of P wave duration/P wave voltage for new-onset AF.

## 5. Conclusion

P wave duration/P wave voltage is a cheap, practical, and reliable electrocardiographic parameter, which can play a promising role for both in predicting and elucidating a mechanism of developing new-onset AF. Moreover, large-scale clinical studies with more patient numbers are needed to determine an important link between P wave duration/P wave voltage and pathophysiology of new-onset AF.

## Figures and Tables

**Table 1:** Demographic characteristics.

	New-onset atrial fibrillation (*n* = 150)	Nonatrial fibrillation (*n* = 490)	*p* value
Age (years)	68.3 ± 7.9	66.9 ± 6.7	0.149
Male sex (%)	54 (36.0)	201 (41.0)	0.272
BMI (kg/m^2^)	28.3 ± 8.2	28.8 ± 7.3	0.893
Diabetes mellitus (%)	50 (33.3)	196 (40.0)	0.142
Smoking (%)	67 (44.7)	147 (30.0)	0.001
Hypertension (%)	122 (81.3)	373 (76.1)	0.182
Hyperlipidemia (%)	15 (10.0)	32 (6.5)	0.154
Prior TIA or stroke (%)	9 (6.0)	20 (4.1)	0.323
Prior diagnosis of CHF (%)	14 (9.3)	32 (6.5)	0.245
Known coronary artery disease (%)	65 (43.3)	199 (40.6)	0.554
Peripheral vascular disease (%)	6 (4.0)	59 (12.0)	0.004
COPD (%)	34 (22.7)	126 (25.7)	0.451
CHA_2_DS_2_VASc score	3.4 ± 1.3	3.2 ± 1.4	0.181

Expressed as mean ± SD and *N* (%) for continuous and categorical variables. BMI: body mass index; CHA_2_DS_2_VASc: congestive heart failure-hypertension-age ≥ 75 years-diabetes mellitus-stroke-vascular disease-age 65–74 years-sex; CHF: congestive heart failure; COPD: chronic obstructive pulmonary disease; TIA: transient ischemic attack.

**Table 2:** Echocardiographic parameters of the patients.

	New-onset atrial fibrillation (*n* = 150)	Nonatrial fibrillation (*n* = 490)	*p* value
Ejection fraction (mean %)	54.6 ± 11.0	54.2 ± 11.3	0.819
LA diameter (mm)	39.6 ± 1.8	39.1 ± 1.9	0.113
LA volume index (mL/mm^2^)	36.0 ± 1.5	31.2 ± 1.8	<0.001
LV end-diastolic diameter (mm)	48.5 ± 6.0	49.8 ± 5.9	0.541
PAP (mmHg)	37.5 ± 14.1	36.4 ± 14.2	0.425
IVSD (mm)	12.4 ± 1.5	12.3 ± 1.8	0.688
PWD (mm)	12.5 ± 1.7	12.2 ± 1.9	0.490
Mitral regurgitation (%)			
None-mild	135 (90)	430 (87.8)	0.175
Moderate-severe	15 (10)	60 (12.2)	0.223
Aortic regurgitation (%)			
None-mild	145 (96.7)	479 (97.7)	0.756
Moderate-severe	5 (3.3)	11 (2.2)	0.313
Aortic stenosis (%)			
None-mild	148 (98.6)	488 (99.5)	0.922
Moderate-severe	2 (1.4)	2 (0.5)	0.456

Data are presented as mean ± SD or *n* (%). Valvular stenosis and regurgitations were graded according to the current guidelines. [13] IVSD: interventricular septal diameter; LA: left atrium; LV: left ventricle; PAP: pulmonary artery systolic pressure; PWD: posterior wall diameter.

**Table 3:** Laboratory findings.

	New-onset atrial fibrillation (*n* = 150)	Nonatrial fibrillation (*n* = 490)	*p* value
WBC (×10^3^/L)	7.7 ± 1.9	8.0 ± 2.0	0.103
Hemoglobin (g/dL)	13.5 ± 1.5	13.3 ± 1.5	0.398
Platelet count (×10^3^/L)	247.6 ± 58.6	246.4 ± 62.7	0.821
Glucose (mg/dL)	110.0 ± 44.6	112.9 ± 46.7	0.279
Creatinine (mg/dL)	0.9 ± 0.2	0.9 ± 0.2	0.991
Total cholesterol (mg/dL)	205.0 ± 41.3	201.0 ± 42.0	0.390
LDL cholesterol (mg/dL)	125.6 ± 32.1	122.7 ± 32.6	0.384
HDL cholesterol (mg/dL)	46.9 ± 9.5	45.0 ± 9.9	0.010
Triglyceride (mg/dL)	163.0 ± 77.4	158.7 ± 79.0	0.418
AST (U/L)	19.8 ± 7.2	19.6 ± 7.3	0.824
ALT (U/L)	16.9 ± 7.0	17.5 ± 7.4	0.399
TSH (*μ*U/mL)	2.7 ± 1.2	2.7 ± 1.2	0.791
CRP (pg/mL)	2.3 ± 1.0	1.5 ± 1.0	<0.001
BNP (pg/mL)	92.3 ± 56.4	62.8 ± 40.6	<0.001

Data are presented as mean ± SD. ALT: alanine aminotransferase; AST: aspartate aminotransferase; BNP: brain natriuretic peptide; CRP: C-reactive protein; HDL: high-density lipoprotein; LDL: low-density lipoprotein; TSH: thyroid-stimulating hormone; WBC: white blood cell.

**Table 4:** 24-hour Holter findings.

	New-onset atrial fibrillation (*n* = 150)	Nonatrial fibrillation (*n* = 490)	*p* value
No. of PACs per hour	3.1 (2.3–3.8)	2.8 (2.1–3.6)	0.032
No. of PVCs per hour	1.4 (0.8–4.2)	1.2 (0.6–4.2)	0.070
Mean heart rate (BPM)	71.0 ± 7.4	71.1 ± 7.9	0.999
Maximal heart rate (BPM)	137.1 ± 9.6	136.3 ± 9.7	0.509
Minimal heart rate (BPM)	46.2 ± 5.7	45.9 ± 5.2	0.879

Data are presented as median (interquartile range) or mean ± SD. BPM: beats per minute; PAC: premature atrial contraction; PVC: premature ventricular contraction.

**Table 5:** Medications taken by all patients.

	New-onset atrial fibrillation (*n* = 150)	Nonatrial fibrillation (*n* = 490)	*p* value
ASA (%)	97 (64.7)	346 (70.6)	0.167
ACE inhibitors/ARB (%)	111 (74.0)	339 (69.2)	0.259
Beta blockers (%)	74 (49.3)	228 (46.5)	0.547
CA channel blockers (%)	48 (32.0)	191 (39.0)	0.122
OAD (%)	49 (32.7)	193 (39.4)	0.137
Insulin (%)	17 (11.3)	61 (12.4)	0.715
Statins (%)	37 (24.7)	147 (30.0)	0.207
MRA (%)	13 (8.7)	36 (7.3)	0.595
Diuretics (%)	17 (11.3)	42 (8.6)	0.306

Data are presented as *n* (%). ACE: angiotensin-converting enzyme; ARB: angiotensin receptor blocker; ASA: acetylsalicylic acid; CA: calcium; OAD: oral antidiabetic; MRA: mineralocorticoid receptor antagonists.

**Table 6:** Electrocardiographic findings of the study population.

	New-onset atrial fibrillation (*n* = 150)	Nonatrial fibrillation (*n* = 490)	*p* value
P wave duration (ms)	123.2 ± 12.8	119.3 ± 17.3	0.024
<120 ms (%)	62 (41.3)	229 (46.7)	0.245
120–140 ms (%)	72 (48.0)	215 (44.0)	0.374
>140 ms (%)	16 (10.7)	46 (9.3)	0.643
P wave voltage (mV)	0.12 ± 0.04	0.13 ± 0.04	<0.001
>0.20 mV (%)	4 (2.7)	23 (4.7)	0.280
0.10–0.20 mV (%)	102 (68.0)	304 (62.0)	0.185
<0.10 mV (%)	44 (29.3)	163 (33.3)	0.344
P wave duration/P wave voltage (ms/mV)	1284.7 ± 508.0	924.1 ± 462.0	<0.001

Data are expressed as mean ± SD and N (%) for continuous and categorical variables.

**Table 7:** Univariate and multivariate regression analyses of predictors of new-onset atrial fibrillation.

Variables	Univariate analysis		Multivariate analysis	
	Odds ratio (95% CI)	*p*	Odds ratio (95% CI)	*p*
Age (years)	1.027 (1.002–1.054)	0.038	1.029 (0.976–1.086)	0.285
Gender	1.236 (0.846–1.806)	0.272		
Smoking	1.884 (1.294–2.742)	0.001	4.008 (1.707–9.409)	0.001
CHA_2_DS_2_VASc	1.095 (0.961–1.247)	0.172		
CRP (pg/mL)	1.869 (1.569–2.226)	<0.001	0.925 (0.412–2.075)	0.850
BNP (pg/mL)	1.013 (1.009–1.018)	<0.001	1.000 (0.981–1.019)	0.967
Ejection fraction	1.004 (0.987–1.020)	0.665		
LA diameter (mm)	1.126 (1.024–1.237)	0.014	1.084 (0.869–1.352)	0.474
LA volume index (mL/m^2^)	7.874 (4.850–12.784)	<0.001	7.108 (4.400–11.483)	<0.001
P wave duration (ms)	1.015 (1.003–1.026)	0.011	1.014 (0.989–1.040)	0.264
P wave voltage (mV)	0.949 (0.914–0.985)	0.006	1.157 (0.982–1.445)	0.121
P wave duration/P wave voltage (ms/mV)	1.001 (1.001–1.002)	<0.001	1.002 (1.000–1.003)	0.044
PAC	1.136 (0.987–1.307)	0.075		

CHA_2_DS_2_VASc: congestive heart failure-hypertension-age≥75 years-diabetes mellitus-stroke-vascular disease-age 65–74 years-sex; BNP: brain natriuretic peptide; CRP: C-reactive protein; LA: left atrium; PAC: premature atrial contraction.

## Data Availability

The data used to support the findings of this study are available from the corresponding author upon request.
